# Supplementary material for: Bayesian hierarchical meta‐analytic methods for modeling surrogate relationships that vary across treatment classes using aggregate data
Source: Stat Med. 2020 Jan 28;39(8):1103–24. doi: 10.1002/sim.8465 (PMC7065251; doi:10.1002/sim.8465)
Supplement: Supplementary file 1 — Data S1 Supporting Information [file SIM-39-1103-s001.pdf]

# Supplementary material

## A Bootstrapping method

A bootstrapping method was used to estimate the within-study correlations $\rho_{wi}$ between the treatment effects on the surrogate and the final outcome by drawing 5000 bootstrap samples with replacement from the IPD. The treatment effects (logHR or logOR) on all outcomes (TR, PFS and OS) were estimated for each bootstrap sample by fitting Cox regression to data on PFS and OS and a logistic regression to data on TR. The Pearson correlation coefficient between the treatment effects (logHR or logOR) were obtained and used as a measure of association for the two pairs of outcomes: TR-PFS and PFS-OS.

Table 1 presents the within-study correlations between treatment effects on each pair of outcomes for each of the treatment classes.

Table 1: Within-study correlations

| | **Endpoints** | |
|---|---|---|
| Classes of treatment | PFS-OS | TR-PFS |
| chemotherapy | 0.561 | -0.413 |
| anti-EGFR | 0.513 | -0.433 |
| anti-Angiogenic | 0.535 | -0.302 |

# B Tables for the performance of $\hat{\lambda}_{1j}$ for all different scenarios within treatment classes and across methods

## B.1 1st scenario

Table 2: Performance of $\hat{\lambda}_{1j}$ for 1st scenario

| Methods | Coverage probability$_j$ (Mean) | Absolute Bias (Mean) | RMSE$_j$ | Width Ratio$_j$ (Mean) | MCE$_j$ | Probability of strong association$_j$ (Mean) |
|---|---|---|---|---|---|---|
| **subgroup analysis** | | | | | | |
| $1^{st}$ treatment class | 0.95 | 0.07 | 0.08 | | 0.003 | 0.80 |
| $2^{nd}$ treatment class | 0.95 | 0.07 | 0.10 | | 0.003 | 0.79 |
| $3^{rd}$ treatment class | 0.96 | 0.07 | 0.10 | | 0.002 | 0.81 |
| $4^{th}$ treatment class | 0.97 | 0.08 | 0.10 | | 0.002 | 0.81 |
| $5^{th}$ treatment class | 0.94 | 0.08 | 0.10 | | 0.003 | 0.81 |
| **F-EX model** | | | | | | |
| $1^{st}$ treatment class | 0.94 | 0.06 | 0.07 | 0.77 | 0.002 | 0.85 |
| $2^{nd}$ treatment class | 0.97 | 0.05 | 0.06 | 0.71 | 0.002 | 0.84 |
| $3^{rd}$ treatment class | 0.98 | 0.05 | 0.06 | 0.70 | 0.002 | 0.85 |
| $4^{th}$ treatment class | 0.97 | 0.05 | 0.07 | 0.69 | 0.002 | 0.84 |
| $5^{th}$ treatment class | 0.90 | 0.07 | 0.09 | 0.72 | 0.003 | 0.83 |
| **P-EX** | | | | | | |
| $1^{st}$ treatment class | 0.94 | 0.06 | 0.07 | 0.78 | 0.002 | 0.85 |
| $2^{st}$ treatment class | 0.97 | 0.05 | 0.06 | 0.72 | 0.002 | 0.85 |
| $3^{st}$ treatment class | 0.98 | 0.05 | 0.06 | 0.70 | 0.002 | 0.84 |
| $4^{st}$ treatment class | 0.97 | 0.05 | 0.07 | 0.70 | 0.002 | 0.84 |
| $5^{st}$ treatment class | 0.91 | 0.07 | 0.09 | 0.72 | 0.003 | 0.84 |

## B.2 2nd scenario

Table 3: Performance of $\hat{\lambda}_{1j}$ for 2nd scenario

| Methods | Coverage probability$_j$ (Mean) | Absolute Bias (Mean) | RMSE$_j$ | Width Ratio$_j$ (Mean) | MCE$_j$ | Probability of strong association$_j$ (Mean) |
|---|---|---|---|---|---|---|
| **subgroup analysis** | | | | | | |
| $1^{st}$ treatment class | 0.98 | 0.10 | 0.13 | | 0.005 | 0.68 |
| $2^{nd}$ treatment class | 0.98 | 0.11 | 0.14 | | 0.005 | 0.66 |
| $3^{rd}$ treatment class | 0.98 | 0.11 | 0.15 | | 0.004 | 0.70 |
| $4^{th}$ treatment class | 0.98 | 0.11 | 0.15 | | 0.005 | 0.75 |
| $5^{th}$ treatment class | 0.98 | 0.11 | 0.16 | | 0.005 | 0.78 |
| **F-EX model** | | | | | | |
| $1^{st}$ treatment class | 0.97 | 0.07 | 0.09 | 0.64 | 0.003 | 0.90 |
| $2^{nd}$ treatment class | 0.98 | 0.06 | 0.08 | 0.60 | 0.003 | 0.90 |
| $3^{rd}$ treatment class | 0.98 | 0.06 | 0.08 | 0.59 | 0.003 | 0.90 |
| $4^{th}$ treatment class | 0.98 | 0.07 | 0.08 | 0.59 | 0.003 | 0.90 |
| $5^{th}$ treatment class | 0.94 | 0.09 | 0.10 | 0.59 | 0.003 | 0.90 |
| **P-EX** | | | | | | |
| $1^{st}$ treatment class | 0.98 | 0.07 | 0.09 | 0.65 | 0.003 | 0.90 |
| $2^{st}$ treatment class | 0.98 | 0.06 | 0.08 | 0.61 | 0.003 | 0.90 |
| $3^{st}$ treatment class | 0.99 | 0.06 | 0.08 | 0.60 | 0.003 | 0.90 |
| $4^{st}$ treatment class | 0.98 | 0.07 | 0.08 | 0.59 | 0.003 | 0.90 |
| $5^{st}$ treatment class | 0.94 | 0.09 | 0.11 | 0.60 | 0.004 | 0.90 |

## B.3 3rd scenario

Table 4: Performance of $\hat{\lambda}_{1j}$ for 3rd scenario

| Methods | Coverage probability$_j$ (Mean) | Absolute Bias (Mean) | RMSE$_j$ | Width Ratio$_j$ (Mean) | MCE$_j$ | Probability of strong association$_j$ (Mean) |
|---|---|---|---|---|---|---|
| **subgroup analysis** | | | | | | |
| $1^{st}$ treatment class | 1.00 | 0.19 | 0.27 | | 0.038 | 0.03 |
| $2^{nd}$ treatment class | 0.99 | 0.10 | 0.14 | | 0.005 | 0.71 |
| $3^{rd}$ treatment class | 0.99 | 0.14 | 0.18 | | 0.028 | 0.52 |
| $4^{th}$ treatment class | 0.98 | 0.10 | 0.13 | | 0.003 | 0.81 |
| $5^{th}$ treatment class | 0.97 | 0.13 | 0.19 | | 0.011 | 0.72 |
| **F-EX model** | | | | | | |
| $1^{st}$ treatment class | 0.99 | 0.08 | 0.10 | 0.26 | 0.004 | 0.84 |
| $2^{nd}$ treatment class | 0.99 | 0.06 | 0.08 | 0.63 | 0.003 | 0.90 |
| $3^{rd}$ treatment class | 0.99 | 0.07 | 0.08 | 0.48 | 0.004 | 0.90 |
| $4^{th}$ treatment class | 0.98 | 0.08 | 0.08 | 0.69 | 0.003 | 0.88 |
| $5^{th}$ treatment class | 0.97 | 0.08 | 0.10 | 0.55 | 0.003 | 0.91 |
| **P-EX** | | | | | | |
| $1^{st}$ treatment class | 0.99 | 0.08 | 0.10 | 0.27 | 0.004 | 0.83 |
| $2^{st}$ treatment class | 0.99 | 0.06 | 0.08 | 0.64 | 0.003 | 0.90 |
| $3^{st}$ treatment class | 0.99 | 0.06 | 0.08 | 0.49 | 0.004 | 0.90 |
| $4^{st}$ treatment class | 0.98 | 0.07 | 0.09 | 0.69 | 0.003 | 0.88 |
| $5^{st}$ treatment class | 0.97 | 0.08 | 0.10 | 0.56 | 0.004 | 0.92 |

## B.4 4th scenario

Table 5: Performance of $\hat{\lambda}_{1j}$ for 4th scenario

| Methods | Coverage probability$_j$ (Mean) | Absolute Bias (Mean) | RMSE$_j$ | Width Ratio$_j$ (Mean) | MCE$_j$ | Probability of strong association$_j$ (Mean) |
|---|---|---|---|---|---|---|
| **subgroup analysis** | | | | | | |
| $1^{st}$ treatment class | 0.96 | 0.08 | 0.10 | | 0.004 | 0.85 |
| $2^{nd}$ treatment class | 0.93 | 0.09 | 0.11 | | 0.004 | 0.90 |
| $3^{rd}$ treatment class | 0.94 | 0.09 | 0.11 | | 0.004 | 0.91 |
| $4^{th}$ treatment class | 0.95 | 0.09 | 0.11 | | 0.004 | 0.90 |
| $5^{th}$ treatment class | 0.93 | 0.10 | 0.12 | | 0.004 | 0.90 |
| **F-EX model** | | | | | | |
| $1^{st}$ treatment class | 0.96 | 0.07 | 0.09 | 0.93 | 0.004 | 0.88 |
| $2^{nd}$ treatment class | 0.94 | 0.08 | 0.10 | 0.89 | 0.004 | 0.91 |
| $3^{rd}$ treatment class | 0.93 | 0.08 | 0.10 | 0.89 | 0.004 | 0.93 |
| $4^{th}$ treatment class | 0.94 | 0.08 | 0.10 | 0.90 | 0.004 | 0.92 |
| $5^{th}$ treatment class | 0.93 | 0.09 | 0.11 | 0.90 | 0.004 | 0.91 |
| **P-EX** | | | | | | |
| $1^{st}$ treatment class | 0.96 | 0.07 | 0.09 | 0.92 | 0.003 | 0.89 |
| $2^{st}$ treatment class | 0.96 | 0.07 | 0.09 | 0.84 | 0.003 | 0.92 |
| $3^{st}$ treatment class | 0.95 | 0.07 | 0.09 | 0.83 | 0.003 | 0.93 |
| $4^{st}$ treatment class | 0.94 | 0.07 | 0.09 | 0.83 | 0.003 | 0.92 |
| $5^{st}$ treatment class | 0.90 | 0.09 | 0.10 | 0.84 | 0.003 | 0.91 |

## B.5 5th scenario

Table 6: Performance of $\hat{\lambda}_{1j}$ for 5th scenario

| Methods | Coverage probability$_j$ (Mean) | Absolute Bias (Mean) | RMSE$_j$ | Width Ratio$_j$ (Mean) | MCE$_j$ | Probability of strong association$_j$ (Mean) |
|---|---|---|---|---|---|---|
| **subgroup analysis** | | | | | | |
| $1^{st}$ treatment class | 0.98 | 0.13 | 0.16 | | 0.005 | 0.85 |
| $2^{nd}$ treatment class | 0.96 | 0.13 | 0.17 | | 0.005 | 0.90 |
| $3^{rd}$ treatment class | 0.96 | 0.13 | 0.17 | | 0.005 | 0.91 |
| $4^{th}$ treatment class | 0.98 | 0.14 | 0.16 | | 0.005 | 0.90 |
| $5^{th}$ treatment class | 0.97 | 0.14 | 0.17 | | 0.005 | 0.90 |
| **F-EX model** | | | | | | |
| $1^{st}$ treatment class | 0.96 | 0.14 | 0.19 | 1.06 | 0.005 | 0.90 |
| $2^{nd}$ treatment class | 0.96 | 0.11 | 0.14 | 0.80 | 0.005 | 0.94 |
| $3^{rd}$ treatment class | 0.96 | 0.11 | 0.14 | 0.80 | 0.005 | 0.92 |
| $4^{th}$ treatment class | 0.98 | 0.11 | 0.14 | 0.81 | 0.005 | 0.92 |
| $5^{th}$ treatment class | 0.96 | 0.12 | 0.15 | 0.80 | 0.005 | 0.91 |
| **P-EX** | | | | | | |
| $1^{st}$ treatment class | 0.98 | 0.10 | 0.14 | 0.90 | 0.004 | 0.88 |
| $2^{st}$ treatment class | 0.98 | 0.09 | 0.11 | 0.76 | 0.004 | 0.94 |
| $3^{st}$ treatment class | 0.97 | 0.09 | 0.11 | 0.74 | 0.004 | 0.93 |
| $4^{st}$ treatment class | 0.97 | 0.10 | 0.12 | 0.75 | 0.004 | 0.92 |
| $5^{st}$ treatment class | 0.95 | 0.11 | 0.13 | 0.74 | 0.004 | 0.92 |

## B.6 6th scenario

Table 7: Performance of $\hat{\lambda}_{1j}$ for 6th scenario

| Methods | Coverage probability$_j$ (Mean) | Absolute Bias (Mean) | RMSE$_j$ | Width Ratio$_j$ (Mean) | MCE$_j$ | Probability of strong association$_j$ (Mean) |
|---|---|---|---|---|---|---|
| **subgroup analysis** | | | | | | |
| $1^{st}$ treatment class | 1.00 | 0.22 | 0.31 | | 0.045 | 0.07 |
| $2^{nd}$ treatment class | 0.97 | 0.12 | 0.15 | | 0.015 | 0.90 |
| $3^{rd}$ treatment class | 0.98 | 0.15 | 0.20 | | 0.025 | 0.86 |
| $4^{th}$ treatment class | 0.97 | 0.11 | 0.14 | | 0.005 | 0.90 |
| $5^{th}$ treatment class | 0.98 | 0.14 | 0.20 | | 0.018 | 0.90 |
| **F-EX model** | | | | | | |
| $1^{st}$ treatment class | 0.91 | 0.46 | 0.51 | 0.58 | 0.005 | 0.71 |
| $2^{nd}$ treatment class | 0.98 | 0.08 | 0.11 | 0.76 | 0.005 | 0.93 |
| $3^{rd}$ treatment class | 0.98 | 0.10 | 0.13 | 0.64 | 0.005 | 0.92 |
| $4^{th}$ treatment class | 0.95 | 0.10 | 0.12 | 0.82 | 0.005 | 0.93 |
| $5^{th}$ treatment class | 0.96 | 0.12 | 0.15 | 0.70 | 0.005 | 0.92 |
| **P-EX** | | | | | | |
| $1^{st}$ treatment class | 0.96 | 0.34 | 0.40 | 0.62 | 0.005 | 0.68 |
| $2^{st}$ treatment class | 0.98 | 0.08 | 0.10 | 0.75 | 0.005 | 0.93 |
| $3^{st}$ treatment class | 0.99 | 0.09 | 0.13 | 0.62 | 0.005 | 0.92 |
| $4^{st}$ treatment class | 0.95 | 0.09 | 0.12 | 0.81 | 0.005 | 0.92 |
| $5^{st}$ treatment class | 0.96 | 0.12 | 0.14 | 0.70 | 0.005 | 0.92 |

### B.7 7th scenario

Table 8: Performance of $\hat{\lambda}_{1j}$ for 7th scenario

| Methods | Coverage probability$_j$ (Mean) | Absolute Bias (Mean) | RMSE$_j$ | Width Ratio$_j$ (Mean) | MCE$_j$ |
|---|---|---|---|---|---|
| **subgroup analysis** | | | | | |
| $1^{st}$ treatment class | 0.96 | 0.07 | 0.09 | | 0.002 |
| $2^{nd}$ treatment class | 0.96 | 0.14 | 0.18 | | 0.003 |
| $3^{rd}$ treatment class | 0.96 | 0.08 | 0.11 | | 0.003 |
| $4^{th}$ treatment class | 0.95 | 0.16 | 0.20 | | 0.003 |
| $5^{th}$ treatment class | 0.94 | 0.09 | 0.12 | | 0.003 |
| **F-EX model** | | | | | |
| $1^{st}$ treatment class | 0.95 | 0.07 | 0.08 | 0.91 | 0.002 |
| $2^{nd}$ treatment class | 0.98 | 0.08 | 0.11 | 0.71 | 0.002 |
| $3^{rd}$ treatment class | 0.97 | 0.06 | 0.08 | 0.80 | 0.002 |
| $4^{th}$ treatment class | 0.97 | 0.10 | 0.13 | 0.68 | 0.003 |
| $5^{th}$ treatment class | 0.89 | 0.10 | 0.13 | 0.86 | 0.002 |
| **P-EX** | | | | | |
| $1^{st}$ treatment class | 0.95 | 0.07 | 0.08 | 0.91 | 0.002 |
| $2^{st}$ treatment class | 0.98 | 0.08 | 0.11 | 0.71 | 0.003 |
| $3^{st}$ treatment class | 0.97 | 0.06 | 0.08 | 0.81 | 0.002 |
| $4^{st}$ treatment class | 0.97 | 0.10 | 0.13 | 0.69 | 0.003 |
| $5^{st}$ treatment class | 0.88 | 0.10 | 0.12 | 0.86 | 0.002 |

## B.8 8th scenario

Table 9: Performance of $\hat{\lambda}_{1j}$ for 8th scenario

| Methods | Coverage probability$_j$ (Mean) | Absolute Bias (Mean) | RMSE$_j$ | Width Ratio$_j$ (Mean) | MCE$_j$ |
|---|---|---|---|---|---|
| **subgroup analysis** | | | | | |
| $1^{st}$ treatment class | 0.98 | 0.11 | 0.14 | | 0.004 |
| $2^{nd}$ treatment class | 0.96 | 0.21 | 0.27 | | 0.008 |
| $3^{rd}$ treatment class | 0.97 | 0.13 | 0.17 | | 0.005 |
| $4^{th}$ treatment class | 0.96 | 0.24 | 0.31 | | 0.008 |
| $5^{th}$ treatment class | 0.97 | 0.13 | 0.18 | | 0.005 |
| **F-EX model** | | | | | |
| $1^{st}$ treatment class | 0.97 | 0.09 | 0.11 | 0.78 | 0.003 |
| $2^{nd}$ treatment class | 0.98 | 0.11 | 0.14 | 0.59 | 0.004 |
| $3^{rd}$ treatment class | 0.98 | 0.08 | 0.11 | 0.68 | 0.003 |
| $4^{th}$ treatment class | 0.98 | 0.13 | 0.16 | 0.56 | 0.005 |
| $5^{th}$ treatment class | 0.90 | 0.13 | 0.11 | 0.71 | 0.003 |
| **P-EX** | | | | | |
| $1^{st}$ treatment class | 0.97 | 0.09 | 0.11 | 0.78 | 0.003 |
| $2^{st}$ treatment class | 0.98 | 0.11 | 0.14 | 0.60 | 0.005 |
| $3^{st}$ treatment class | 0.98 | 0.08 | 0.11 | 0.68 | 0.003 |
| $4^{st}$ treatment class | 0.98 | 0.13 | 0.16 | 0.57 | 0.005 |
| $5^{st}$ treatment class | 0.90 | 0.13 | 0.11 | 0.72 | 0.003 |

## B.9 9th scenario

Table 10: Performance of $\hat{\lambda}_{1j}$ for 9th scenario

| Methods | Coverage probability$_j$ (Mean) | Absolute Bias (Mean) | RMSE$_j$ | Width Ratio$_j$ (Mean) | MCE$_j$ |
|---|---|---|---|---|---|
| **subgroup analysis** | | | | | |
| $1^{st}$ treatment class | 1.00 | 0.20 | 0.14 | | 0.045 |
| $2^{nd}$ treatment class | 0.96 | 0.21 | 0.27 | | 0.015 |
| $3^{rd}$ treatment class | 0.99 | 0.16 | 0.17 | | 0.025 |
| $4^{th}$ treatment class | 0.96 | 0.21 | 0.31 | | 0.005 |
| $5^{th}$ treatment class | 0.98 | 0.15 | 0.18 | | 0.018 |
| **F-EX model** | | | | | |
| $1^{st}$ treatment class | 0.98 | 0.13 | 0.15 | 0.34 | 0.005 |
| $2^{nd}$ treatment class | 0.98 | 0.12 | 0.15 | 0.61 | 0.005 |
| $3^{rd}$ treatment class | 0.99 | 0.09 | 0.11 | 0.56 | 0.003 |
| $4^{th}$ treatment class | 0.98 | 0.12 | 0.16 | 0.63 | 0.005 |
| $5^{th}$ treatment class | 0.93 | 0.13 | 0.15 | 0.56 | 0.003 |
| **P-EX** | | | | | |
| $1^{st}$ treatment class | 0.99 | 0.13 | 0.15 | 0.35 | 0.005 |
| $2^{st}$ treatment class | 0.98 | 0.12 | 0.15 | 0.62 | 0.005 |
| $3^{st}$ treatment class | 0.99 | 0.09 | 0.11 | 0.57 | 0.004 |
| $4^{st}$ treatment class | 0.97 | 0.13 | 0.16 | 0.64 | 0.005 |
| $5^{st}$ treatment class | 0.94 | 0.13 | 0.15 | 0.67 | 0.003 |

# C Tables for the performance of $\hat{\mu}_{2ij}$ for all different scenarios within treatment classes and across methods

## C.1 1st scenario

Table 11: Performance of predictions $\hat{\mu}_{2ij}$ for 1st scenario

| Methods | Coverage probability$_j$ (Mean) | Absolute Bias (Mean) | RMSE$_j$ | Width Ratio$_j$ (Mean) | MCE$_j$ |
|---|---|---|---|---|---|
| **Subgroup analysis** | | | | | |
| $1^{st}$ treatment class | 0.95 | 0.09 | 0.10 | | 0.003 |
| $2^{nd}$ treatment class | 0.95 | 0.09 | 0.10 | | 0.003 |
| $3^{rd}$ treatment class | 0.95 | 0.09 | 0.11 | | 0.003 |
| $4^{th}$ treatment class | 0.95 | 0.09 | 0.11 | | 0.003 |
| $5^{th}$ treatment class | 0.95 | 0.09 | 0.11 | | 0.003 |
| **F-EX model** | | | | | |
| $1^{st}$ treatment class | 0.95 | 0.08 | 0.10 | 0.96 | 0.002 |
| $2^{nd}$ treatment class | 0.95 | 0.08 | 0.10 | 0.93 | 0.002 |
| $3^{rd}$ treatment class | 0.95 | 0.08 | 0.10 | 0.92 | 0.002 |
| $4^{th}$ treatment class | 0.95 | 0.08 | 0.10 | 0.92 | 0.002 |
| $5^{th}$ treatment class | 0.95 | 0.08 | 0.10 | 0.93 | 0.002 |
| **P-EX model** | | | | | |
| $1^{st}$ treatment class | 0.95 | 0.08 | 0.10 | 0.95 | 0.002 |
| $2^{nd}$ treatment class | 0.95 | 0.08 | 0.10 | 0.93 | 0.002 |
| $3^{rd}$ treatment class | 0.95 | 0.08 | 0.10 | 0.93 | 0.002 |
| $4^{th}$ treatment class | 0.95 | 0.08 | 0.10 | 0.92 | 0.002 |
| $5^{th}$ treatment class | 0.95 | 0.08 | 0.10 | 0.93 | 0.002 |

## C.2 2nd scenario

Table 12: Performance of predictions $\hat{\mu}_{2ij}$ for 2nd scenario

| Methods | Coverage probability$_j$ (Mean) | Absolute Bias (Mean) | RMSE$_j$ | Width Ratio$_j$ (Mean) | MCE$_j$ |
|---|---|---|---|---|---|
| **Subgroup analysis** | | | | | |
| $1^{st}$ treatment class | 0.98 | 0.10 | 0.12 | | 0.009 |
| $2^{nd}$ treatment class | 0.98 | 0.11 | 0.12 | | 0.009 |
| $3^{rd}$ treatment class | 0.98 | 0.11 | 0.13 | | 0.009 |
| $4^{th}$ treatment class | 0.98 | 0.11 | 0.13 | | 0.010 |
| $5^{th}$ treatment class | 0.98 | 0.11 | 0.13 | | 0.010 |
| **F-EX model** | | | | | |
| $1^{st}$ treatment class | 0.98 | 0.08 | 0.10 | 0.81 | 0.004 |
| $2^{nd}$ treatment class | 0.98 | 0.08 | 0.10 | 0.79 | 0.004 |
| $3^{rd}$ treatment class | 0.98 | 0.08 | 0.10 | 0.79 | 0.004 |
| $4^{th}$ treatment class | 0.98 | 0.08 | 0.10 | 0.79 | 0.004 |
| $5^{th}$ treatment class | 0.98 | 0.09 | 0.11 | 0.80 | 0.004 |
| **P-EX model** | | | | | |
| $1^{st}$ treatment class | 0.98 | 0.08 | 0.10 | 0.82 | 0.004 |
| $2^{nd}$ treatment class | 0.98 | 0.08 | 0.10 | 0.80 | 0.004 |
| $3^{rd}$ treatment class | 0.98 | 0.08 | 0.10 | 0.79 | 0.004 |
| $4^{th}$ treatment class | 0.98 | 0.08 | 0.10 | 0.79 | 0.004 |
| $5^{th}$ treatment class | 0.98 | 0.09 | 0.11 | 0.80 | 0.004 |

### C.3 3rd scenario

Table 13: Performance of predictions $\hat{\mu}_{2ij}$ for 3rd scenario

| Methods | Coverage probability$_j$ (Mean) | Absolute Bias (Mean) | RMSE$_j$ | Width Ratio$_j$ (Mean) | MCE$_j$ |
|---|---|---|---|---|---|
| **Subgroup analysis** | | | | | |
| $1^{st}$ treatment class | 1.00 | 0.18 | 0.38 | | 0.046 |
| $2^{nd}$ treatment class | 0.99 | 0.10 | 0.12 | | 0.008 |
| $3^{rd}$ treatment class | 0.99 | 0.11 | 0.14 | | 0.025 |
| $4^{th}$ treatment class | 0.98 | 0.09 | 0.12 | | 0.010 |
| $5^{th}$ treatment class | 1.00 | 0.10 | 0.13 | | 0.020 |
| **F-EX model** | | | | | |
| $1^{st}$ treatment class | 1.00 | 0.08 | 0.11 | 0.32 | 0.005 |
| $2^{nd}$ treatment class | 0.99 | 0.09 | 0.10 | 0.81 | 0.005 |
| $3^{rd}$ treatment class | 0.99 | 0.08 | 0.11 | 0.62 | 0.005 |
| $4^{th}$ treatment class | 0.97 | 0.08 | 0.10 | 0.88 | 0.005 |
| $5^{th}$ treatment class | 0.99 | 0.09 | 0.11 | 0.74 | 0.005 |
| **P-EX model** | | | | | |
| $1^{st}$ treatment class | 1.00 | 0.08 | 0.11 | 0.34 | 0.008 |
| $2^{nd}$ treatment class | 0.99 | 0.09 | 0.11 | 0.81 | 0.008 |
| $3^{rd}$ treatment class | 0.99 | 0.09 | 0.11 | 0.62 | 0.008 |
| $4^{th}$ treatment class | 0.97 | 0.09 | 0.11 | 0.88 | 0.008 |
| $5^{th}$ treatment class | 0.99 | 0.09 | 0.11 | 0.74 | 0.008 |

## C.4 4th scenario

Table 14: Performance of predictions $\hat{\mu}_{2ij}$ for 4th scenario

| Methods | Coverage probability$_j$ (Mean) | Absolute Bias (Mean) | RMSE$_j$ | Width Ratio$_j$ (Mean) | MCE$_j$ |
|---|---|---|---|---|---|
| **Subgroup analysis** | | | | | |
| $1^{st}$ treatment class | 0.95 | 0.09 | 0.11 | | 0.009 |
| $2^{nd}$ treatment class | 0.95 | 0.15 | 0.19 | | 0.009 |
| $3^{rd}$ treatment class | 0.95 | 0.15 | 0.19 | | 0.009 |
| $4^{th}$ treatment class | 0.95 | 0.15 | 0.19 | | 0.009 |
| $5^{th}$ treatment class | 0.95 | 0.16 | 0.20 | | 0.009 |
| **F-EX model** | | | | | |
| $1^{st}$ treatment class | 0.95 | 0.08 | 0.11 | 0.98 | 0.008 |
| $2^{nd}$ treatment class | 0.95 | 0.14 | 0.18 | 0.97 | 0.008 |
| $3^{rd}$ treatment class | 0.95 | 0.14 | 0.18 | 0.97 | 0.008 |
| $4^{th}$ treatment class | 0.95 | 0.15 | 0.19 | 0.97 | 0.008 |
| $5^{th}$ treatment class | 0.95 | 0.15 | 0.19 | 0.97 | 0.008 |
| **P-EX model** | | | | | |
| $1^{st}$ treatment class | 0.95 | 0.08 | 0.10 | 0.96 | 0.008 |
| $2^{nd}$ treatment class | 0.96 | 0.14 | 0.18 | 0.96 | 0.008 |
| $3^{rd}$ treatment class | 0.96 | 0.14 | 0.18 | 0.96 | 0.008 |
| $4^{th}$ treatment class | 0.96 | 0.15 | 0.18 | 0.95 | 0.008 |
| $5^{th}$ treatment class | 0.95 | 0.15 | 0.19 | 0.95 | 0.008 |

## C.5 5th scenario

Table 15: Performance of predictions $\hat{\mu}_{2ij}$ for 5th scenario

| Methods | Coverage probability$_j$ (Mean) | Absolute Bias (Mean) | RMSE$_j$ | Width Ratio$_j$ (Mean) | MCE$_j$ |
|---|---|---|---|---|---|
| **Subgroup analysis** | | | | | |
| $1^{st}$ treatment class | 0.99 | 0.10 | 0.13 | | 0.010 |
| $2^{nd}$ treatment class | 0.99 | 0.16 | 0.20 | | 0.010 |
| $3^{rd}$ treatment class | 0.99 | 0.17 | 0.21 | | 0.010 |
| $4^{th}$ treatment class | 0.99 | 0.17 | 0.21 | | 0.010 |
| $5^{th}$ treatment class | 0.99 | 0.17 | 0.22 | | 0.010 |
| **F-EX model** | | | | | |
| $1^{st}$ treatment class | 0.98 | 0.11 | 0.15 | 1.08 | 0.010 |
| $2^{nd}$ treatment class | 0.99 | 0.15 | 0.19 | 0.88 | 0.009 |
| $3^{rd}$ treatment class | 0.98 | 0.15 | 0.19 | 0.88 | 0.008 |
| $4^{th}$ treatment class | 0.99 | 0.16 | 0.20 | 0.88 | 0.008 |
| $5^{th}$ treatment class | 0.98 | 0.16 | 0.20 | 0.88 | 0.008 |
| **P-EX model** | | | | | |
| $1^{st}$ treatment class | 0.98 | 0.10 | 0.10 | 0.93 | 0.008 |
| $2^{nd}$ treatment class | 0.99 | 0.14 | 0.18 | 0.86 | 0.008 |
| $3^{rd}$ treatment class | 0.98 | 0.14 | 0.18 | 0.85 | 0.008 |
| $4^{th}$ treatment class | 0.99 | 0.15 | 0.18 | 0.85 | 0.008 |
| $5^{th}$ treatment class | 0.98 | 0.16 | 0.19 | 0.85 | 0.008 |

## C.6 6th scenario

Table 16: Performance of predictions $\hat{\mu}_{2ij}$ for 6th scenario

| Methods | Coverage probability$_j$ (Mean) | Absolute Bias (Mean) | RMSE$_j$ | Width Ratio$_j$ (Mean) | MCE$_j$ |
|---|---|---|---|---|---|
| **Subgroup analysis** | | | | | |
| $1^{st}$ treatment class | 1.00 | 0.18 | 0.30 | | 0.046 |
| $2^{nd}$ treatment class | 0.99 | 0.17 | 0.20 | | 0.008 |
| $3^{rd}$ treatment class | 0.99 | 0.18 | 0.23 | | 0.025 |
| $4^{th}$ treatment class | 0.98 | 0.17 | 0.20 | | 0.010 |
| $5^{th}$ treatment class | 0.99 | 0.18 | 0.23 | | 0.020 |
| **F-EX model** | | | | | |
| $1^{st}$ treatment class | 0.99 | 0.25 | 0.31 | 0.62 | 0.011 |
| $2^{nd}$ treatment class | 0.99 | 0.15 | 0.19 | 0.88 | 0.009 |
| $3^{rd}$ treatment class | 0.99 | 0.16 | 0.20 | 0.73 | 0.009 |
| $4^{th}$ treatment class | 0.98 | 0.15 | 0.19 | 0.93 | 0.009 |
| $5^{th}$ treatment class | 0.99 | 0.16 | 0.20 | 0.82 | 0.009 |
| **P-EX model** | | | | | |
| $1^{st}$ treatment class | 1.00 | 0.15 | 0.20 | 0.56 | 0.009 |
| $2^{nd}$ treatment class | 0.98 | 0.15 | 0.18 | 0.87 | 0.010 |
| $3^{rd}$ treatment class | 0.99 | 0.15 | 0.19 | 0.71 | 0.010 |
| $4^{th}$ treatment class | 0.98 | 0.15 | 0.19 | 0.91 | 0.010 |
| $5^{th}$ treatment class | 0.99 | 0.16 | 0.19 | 0.80 | 0.009 |

## C.7 7th scenario

Table 17: Performance of predictions $\hat{\mu}_{2ij}$ for 7th scenario

| Methods | Coverage probability$_j$ (Mean) | Absolute Bias (Mean) | RMSE$_j$ | Width Ratio$_j$ (Mean) | MCE$_j$ |
|---|---|---|---|---|---|
| **Subgroup analysis** | | | | | |
| $1^{st}$ treatment class | 0.95 | 0.08 | 0.11 | | 0.003 |
| $2^{nd}$ treatment class | 0.95 | 0.26 | 0.33 | | 0.010 |
| $3^{rd}$ treatment class | 0.95 | 0.09 | 0.12 | | 0.003 |
| $4^{th}$ treatment class | 0.95 | 0.27 | 0.34 | | 0.010 |
| $5^{th}$ treatment class | 0.96 | 0.11 | 0.13 | | 0.003 |
| **F-EX model** | | | | | |
| $1^{st}$ treatment class | 0.95 | 0.08 | 0.11 | 0.99 | 0.002 |
| $2^{nd}$ treatment class | 0.95 | 0.25 | 0.32 | 0.94 | 0.007 |
| $3^{rd}$ treatment class | 0.95 | 0.09 | 0.11 | 0.95 | 0.002 |
| $4^{th}$ treatment class | 0.96 | 0.25 | 0.32 | 0.94 | 0.007 |
| $5^{th}$ treatment class | 0.96 | 0.10 | 0.13 | 0.96 | 0.002 |
| **P-EX model** | | | | | |
| $1^{st}$ treatment class | 0.95 | 0.08 | 0.11 | 0.98 | 0.002 |
| $2^{nd}$ treatment class | 0.95 | 0.25 | 0.32 | 0.95 | 0.007 |
| $3^{rd}$ treatment class | 0.95 | 0.09 | 0.11 | 0.95 | 0.002 |
| $4^{th}$ treatment class | 0.96 | 0.25 | 0.32 | 0.94 | 0.007 |
| $5^{th}$ treatment class | 0.96 | 0.10 | 0.13 | 0.96 | 0.002 |

## C.8 8th scenario

Table 18: Performance of predictions $\hat{\mu}_{2ij}$ for 8th scenario

| Methods | Coverage probability$_j$ (Mean) | Absolute Bias (Mean) | RMSE$_j$ | Width Ratio$_j$ (Mean) | MCE$_j$ |
|---|---|---|---|---|---|
| **Subgroup analysis** | | | | | |
| $1^{st}$ treatment class | 0.98 | 0.10 | 0.12 | | 0.010 |
| $2^{nd}$ treatment class | 0.97 | 0.29 | 0.37 | | 0.025 |
| $3^{rd}$ treatment class | 0.98 | 0.10 | 0.13 | | 0.010 |
| $4^{th}$ treatment class | 0.96 | 0.30 | 0.38 | | 0.025 |
| $5^{th}$ treatment class | 0.98 | 0.12 | 0.15 | | 0.010 |
| **F-EX model** | | | | | |
| $1^{st}$ treatment class | 0.98 | 0.09 | 0.12 | 0.89 | 0.004 |
| $2^{nd}$ treatment class | 0.96 | 0.25 | 0.32 | 0.84 | 0.012 |
| $3^{rd}$ treatment class | 0.98 | 0.09 | 0.12 | 0.84 | 0.004 |
| $4^{th}$ treatment class | 0.96 | 0.26 | 0.32 | 0.83 | 0.012 |
| $5^{th}$ treatment class | 0.98 | 0.10 | 0.13 | 0.86 | 0.004 |
| **P-EX model** | | | | | |
| $1^{st}$ treatment class | 0.98 | 0.09 | 0.12 | 0.89 | 0.004 |
| $2^{nd}$ treatment class | 0.96 | 0.25 | 0.32 | 0.84 | 0.012 |
| $3^{rd}$ treatment class | 0.98 | 0.09 | 0.12 | 0.84 | 0.004 |
| $4^{th}$ treatment class | 0.96 | 0.26 | 0.32 | 0.83 | 0.012 |
| $5^{th}$ treatment class | 0.98 | 0.10 | 0.13 | 0.86 | 0.004 |

## C.9 9th scenario

Table 19: Performance of predictions $\hat{\mu}_{2ij}$ for 9th scenario

| Methods | Coverage probability$_j$ (Mean) | Absolute Bias (Mean) | RMSE$_j$ | Width Ratio$_j$ (Mean) | MCE$_j$ |
|---|---|---|---|---|---|
| **Subgroup analysis** | | | | | |
| $1^{st}$ treatment class | 0.98 | 0.19 | 0.34 | | 0.050 |
| $2^{nd}$ treatment class | 0.97 | 0.29 | 0.36 | | 0.020 |
| $3^{rd}$ treatment class | 0.98 | 0.12 | 0.15 | | 0.015 |
| $4^{th}$ treatment class | 0.96 | 0.28 | 0.35 | | 0.025 |
| $5^{th}$ treatment class | 0.98 | 0.12 | 0.16 | | 0.020 |
| **F-EX model** | | | | | |
| $1^{st}$ treatment class | 1.00 | 0.11 | 0.13 | 0.37 | 0.010 |
| $2^{nd}$ treatment class | 0.96 | 0.25 | 0.32 | 0.85 | 0.010 |
| $3^{rd}$ treatment class | 0.99 | 0.10 | 0.12 | 0.67 | 0.006 |
| $4^{th}$ treatment class | 0.96 | 0.26 | 0.32 | 0.89 | 0.012 |
| $5^{th}$ treatment class | 0.99 | 0.11 | 0.14 | 0.80 | 0.005 |
| **P-EX model** | | | | | |
| $1^{st}$ treatment class | 1.00 | 0.11 | 0.13 | 0.40 | 0.011 |
| $2^{nd}$ treatment class | 0.96 | 0.25 | 0.32 | 0.85 | 0.011 |
| $3^{rd}$ treatment class | 0.99 | 0.10 | 0.12 | 0.67 | 0.006 |
| $4^{th}$ treatment class | 0.96 | 0.26 | 0.32 | 0.89 | 0.012 |
| $5^{th}$ treatment class | 0.99 | 0.11 | 0.14 | 0.80 | 0.004 |

# D Other tables

## D.1 mixture weights $p_j$ of P-EX model

Table 20: Mixture weights $p_j$ across all scenarios

| Scenarios | 1st treatment class | 2nd treatment class | 3rd treatment class | 4th treatment class | 5th treatment class |
|---|---|---|---|---|---|
| $1^{st}$ scenario | 0.99 | 0.99 | 0.99 | 0.99 | 0.99 |
| $2^{nd}$ scenario | 0.99 | 0.99 | 0.99 | 0.99 | 0.99 |
| $3^{rd}$ scenario | 0.99 | 0.99 | 0.99 | 0.99 | 0.99 |
| $4^{th}$ scenario | 0.56 | 0.98 | 0.98 | 0.98 | 0.98 |
| $5^{th}$ scenario | 0.31 | 0.88 | 0.88 | 0.88 | 0.88 |
| $6^{th}$ scenario | 0.80 | 0.68 | 0.98 | 0.98 | 0.98 |
| $7^{th}$ scenario | 0.99 | 0.99 | 0.99 | 0.98 | 0.98 |
| $8^{th}$ scenario | 0.98 | 0.99 | 0.99 | 0.98 | 0.98 |
| $9^{th}$ scenario | 0.98 | 0.98 | 0.98 | 0.98 | 0.98 |

## D.2 Bayes factors of $\psi_j^2$ of each treatment class for PFS-OS and TR-PFS

Table 21: Bayes factors of $\psi_j^2$

| outcomes | subgroup analysis | F-EX | P-EX |
|---|---|---|---|
| PFS-OS | | | |
| chemotherapy | 310.43 | 300.80 | 329.29 |
| anti-EGFR | 25.33 | 18.04 | 16.92 |
| anti-angiogenic | 20.98 | 26.97 | 27.90 |
| | | | |
| TR-PFS | | | |
| chemotherapy | 7.89 | 9.09 | 8.31 |
| anti-EGFR | 14.42 | 14.02 | 13.53 |
| anti-angiogenic | 19.83 | 13.24 | 14.18 |

### D.3 Implementation of F-EX model in BUGS

```
model{
#within study precision matrix
for (i in 1:ns) {
Prec_w[i,1:2,1:2] <- inverse(Sigma[i,1:2,1:2])
#covariance matrix for the i-th study
Sigma[i,1,1]<-pow(se[i,1],2)
Sigma[i,2,2]<-pow(se[i,2],2)
Sigma[i,1,2]<-sqrt(Sigma[i,1,1])*sqrt(Sigma[i,2,2])*rho_w[i]
Sigma[i,2,1]<-sqrt(Sigma[i,1,1])*sqrt(Sigma[i,2,2])*rho_w[i]
}
# Random effects model
for (i in 1:ns) {
y[i,1:2]~dmnorm(mu[i,1:2], Prec_w[i,1:2,1:2])
#  product normal formulation for the between study part:
mu[i,1]~dnorm(0,1.0E-3)
mu[i,2]~dnorm(eta[i,class[i]],prec_fin[class[i]])
for (j in 1:nclass) {
eta[i,j]<-lambda0[j]+lambda1[j]*mu[i,1]
}
}
for (j in 1:nclass) {
lambda0[j]~dnorm(beta1,pr1)
lambda1[j]~dnorm(beta2,pr2)
gam_fin[j]~dnorm(0,2)I(0,)
gam_fin.sq[j]<-gam_fin[j]*gam_fin[j]
prec_fin[j]<-1/gam_fin.sq[j]
}
gamma1~dnorm(0,0.01)I(0,)
gamma.sq1<-pow(gamma1,2)
pr1<-1/gamma.sq1
gamma2~dnorm(0,0.01)I(0,)
gamma.sq2<-pow(gamma2,2)
pr2<-1/gamma.sq2
beta1~dnorm(0,1.0E-3)
beta2~dnorm(0,1.0E-3)
}
```

## D.4 Implementation of P-EX model in BUGS

```
model{
#within study precision matrix
for (i in 1:ns) {
Prec_w[i,1:2,1:2] <- inverse(Sigma[i,1:2,1:2])
Sigma[i,1,1]<-pow(se[i,1],2)
Sigma[i,2,2]<-pow(se[i,2],2)
Sigma[i,1,2]<-sqrt(Sigma[i,1,1])*sqrt(Sigma[i,2,2])*rho_w[i]
Sigma[i,2,1]<-sqrt(Sigma[i,1,1])*sqrt(Sigma[i,2,2])*rho_w[i]
}
# Random effects model
for (i in 1:ns) {
y[i,1:2]~dmnorm(mu[i,1:2], Prec_w[i,1:2,1:2])
mu[i,1]~dnorm(0,1.0E-3)
mu[i,2]~dnorm(eta[i,class[i]],prec_fin[class[i]])
for (j in 1:nclass) {
eta[i,j]<-lambda0[j]+lambda1[j]*mu[i,1]
}}
for (j in 1:nclass) {
lambda0[j]~dnorm(beta1,pr1)
sd[j]~dnorm(0,2)I(0,)
gam_fin.sq[j]<-pow(sd[j],2)
prec_fin[j]<-1/gam_fin.sq[j]
c[j]~dbern(p[j])
#exchangeability branch
l1.branch[j,1]~dnorm(beta2,pr2)
#Non-exchangeability branch
l1.branch[j,2]~dnorm(0,0.001)
##construct partial exchangeability
if_branch[j]<-1+step(-(c[j] - 0.5)) #1 for the exchangeable #2  for the non-exchangeable
lambda1[j]<-l1.branch[j,if_branch[j]]
}
gamma1~dnorm(0,0.01)I(0,)
gamma.sq1<-pow(gamma1,2)
pr1<-1/gamma.sq1
gamma2~dnorm(0,0.01)I(0,)
gamma.sq2<-pow(gamma2,2)
pr2<-1/gamma.sq2
beta1~dnorm(0,1.0E-3)
```

```
beta2~dnorm(0,1.0E-3)}
```
